# Highlight: Prey-Determined Patterns in Cone Snail Venom Evolution

**DOI:** 10.1093/molbev/msae139

**Published:** 2024-08-02

**Authors:** Casey McGrath

Numerous animals employ venom as a biological weapon for predation and defense. These complex cocktails of toxins are injected into prey and play a crucial role in the survival and ecological success of these lineages. Cone snails (Fig. 1) are a diverse group of predatory marine gastropods known for their potent venom. Each of the approximately 850 species of cone snail produces a distinctive venom with a unique blend of conotoxins, which they use to immobilize and kill worms, fish, or mollusks. Cone snails employ distinct hunting strategies, such as “taser-and-tether,” “net,” or “ambush-and-assess,” each of which may be associated with different classes of venom proteins. While the diversity of cone snail venoms has been recognized for some time, the evolutionary forces driving this diversity have remained unclear. In a new study published in*Molecular Biology and Evolution*, a team of researchers led by Thomas Koch and Helena Safavi-Hemami from the University of Utah analyzed the venom profiles and prey preferences of 42 species of cone snails (Koch et al. 2024). Their findings reveal that changes in venom complexity and composition are associated with major shifts in prey type, suggesting that the benefits derived from tailoring the poison to its intended victim are a driving force in venom evolution.

**Fig. 1.**
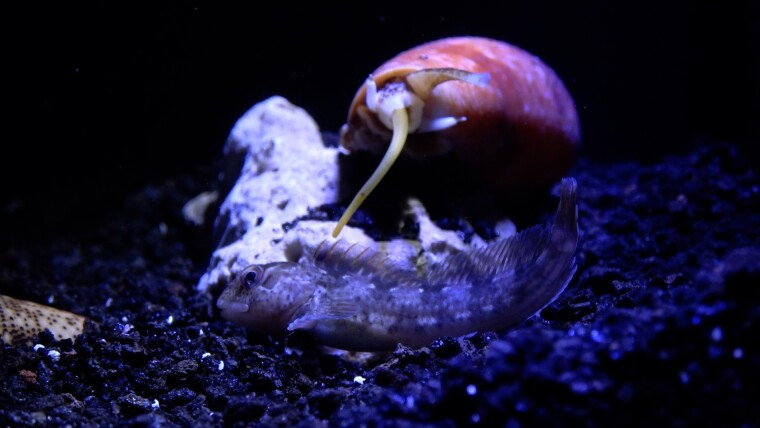
A cone snail hunting a fish. Credit: Dylan Taylor.

To assess the role of prey preferences in venom evolution, the researchers first mapped prey types onto the cone snail family tree. “Cone snails are a perfect model to investigate the hypothesis of prey-targeted toxin evolution because of the well-described diversity of prey they feed on,” says Safavi-Hemami. “Having a venom gland expression dataset of more than 40 species with diverse prey types really allowed us to address this question in a unique way.” Their analysis revealed that the first cone snails likely hunted worms, with three independent origins of fish hunting and a single transition to mollusk hunting in a more recently diverged lineage.

The authors then assessed correlations in the venom composition, prey preference, and expression of conotoxin-encoding genes among the cone snail species. Out of the over 3,400 conotoxin precursors analyzed, the venom of each cone snail species contained between 31 and 236 different conotoxins. Grouping cone snails based on venom composition and conotoxin expression patterns revealed that species with similar prey preferences also tended to have similar venom profiles, as worm-, fish-, and mollusk-hunting snails all formed distinct clusters. This was particularly surprising in the case of fish-hunting snails given the three independent origins of this prey preference. Moreover, species that use “net” and “ambush-and-assess” hunting strategies formed a group that was distinct in terms of venom composition from the “taser-and-tether” hunters, further suggesting a relationship between predation behaviors and conotoxin profiles.

The researchers also compared the conotoxin profiles of juvenile and adult *Conus magus*, a species in which the adults hunt fish while juveniles hunt worms. Consistent with the strong connection between prey type and conotoxin expression, the venom of juvenile *C. magus* grouped with worm-hunting snails while the adult *C. magus* venom was more similar to the venoms of other fish hunters, confirming that the venom in this species changes developmentally along with its prey preference.

Finally, the researchers returned to the lab to assess whether the differences in venom composition that they observed across cone snails with different prey preferences reflected a difference in the effectiveness of the venom on the preferred prey. They injected the venom from two worm hunters, two fish hunters, and two mollusk hunters into goldfish and sea hares (a type of mollusk) and evaluated the effects of each type of venom on the prey animals. Fish injected with the venom of fish hunters were quickly paralyzed or killed, whereas the venom of worm and mollusk hunters had virtually no effect. In contrast, sea hares were impacted by either worm or mollusk hunter venom, whereas the venom of fish-hunting snails had no detectable effect.

Given the multiple independent origins of fish hunting in cone snails, the researchers were surprised at how similar the venoms of the fish-hunting cone snails were. Koch explains, “It appears they have independently arrived at the same optimized solution for capturing fish. This was particularly evident when we injected venom from worm-, snail-, and fish-hunting cone snails into fish, as only the venom from the fish-hunters had a noticeable effect.”

The researchers hope to build upon these results by predicting the molecular targets of the toxins discovered here and by further investigating the molecular mechanisms that gave rise to the venom system of cone snails in the first place. “One potential obstacle in future research is the complexity of these evolutionary transitions, but advanced genomic tools should help us overcome these challenges,” says Koch. “Resolving the cone snail phylogeny has also been notoriously challenging,” he continues. “However, as more cone snail genomes are sequenced, we hope to resolve these phylogenetic uncertainties.”

Such analyses are likely to be of interest not only for evolutionary biologists but also for those developing antivenom therapies and venom-based drug discovery. As with the current study, this will require collaboration among many scientists and institutions. “Over the past few years, we and others have sequenced and published the transcriptomes of many different species with diverse prey types,” notes Safavi-Hemami. “We greatly appreciate that other researchers have shared their data with the scientific community, which enabled us to perform the current study.”

*Want to learn more?* Check out these other recent articles from *Molecular Biology and Evolution* on the evolutionary dynamics of cone snail venom, as well as the relationship between venom and dietary preferences in other model systems.

- “Venom Gene Sequence Diversity and Expression Jointly Shape Diet Adaptation in Pitvipers” (Mason et al. 2022)
- “Comparative Venom Multiomics Reveal the Molecular Mechanisms Driving Adaptation to Diverse Predator–Prey Ecosystems in Closely Related Sea Snakes” (Zheng et al. 2023)
- “Whole Genome Duplication and Gene Evolution in the Hyperdiverse Venomous Gastropods” (Farhat et al. 2023)

## References

[msae139-B1] Farhat S , Modica MV, Puillandre N. Whole genome duplication and gene evolution in the hyperdiverse venomous gastropods. Mol Biol Evol. 2023:40(8):msad171. 10.1093/molbev/msad171. 37494290 PMC10401626

[msae139-B2] Koch TL , Robinson SD, Salcedo PF, Chase K, Biggs J, Fedosov AE, Yandell M, Olivera BM, Safavi-Hemami H. Prey shifts drive venom evolution in cone snails. Mol Biol Evol. 2024:41:msae120. 10.1093/molbev/msae120. PMC11296725 38935574

[msae139-B3] Mason AJ , Holding ML, Rautsaw RM, Rokyta DR, Parkinson CL, Gibbs HL. Venom gene sequence diversity and expression jointly shape diet adaptation in pitvipers. Mol Biol Evol. 2022:39(4):msac082. 10.1093/molbev/msac082. 35413123 PMC9040050

[msae139-B4] Zheng H , Wang J, Fan H, Wang S, Ye R, Li L, Wang S, Li A, Lu Y. Comparative venom multiomics reveal the molecular mechanisms driving adaptation to diverse predator–prey ecosystems in closely related sea snakes. Mol Biol Evol. 2023:40(6):msad125. 10.1093/molbev/msad125. 37279580 PMC10265070

